# A Qualitative Systematic Review of the Barriers and Facilitators of the Reintegration of Men Convicted of a Sexual Offense From Prison or Secure Care into the Community

**DOI:** 10.1177/15248380241254080

**Published:** 2024-05-28

**Authors:** Emma Tuschick, Nikki Carthy, Nadia Wager, Marty Chamberlain

**Affiliations:** 1 School of Social Sciences, Humanities & Law, Teesside University, Middlesbrough, UK

**Keywords:** offenders, sexual assault, sexual abuse, child abuse, anything related to sexual assault, sexual assault, recidivism, sexual assault, prevention, sexual assault

## Abstract

This article is the first qualitative systematic review of studies examining the barriers and facilitators to male sex offenders reintegration from prison or secure care into the community. A search of 16 electronic databases produced 14,218 potential sources, which, after screening, resulted in 79 articles for inclusion. Papers were included if they used qualitative research methods about the barriers, facilitators, perceptions, experiences, and attitudes toward community reintegration from prison or secure care for men convicted of sexual offenses. Included papers were critically appraised and the findings were thematically synthesized. The findings identified that formal and cultural aspects of reintegration, such as probation services, stigmatization, and registration, were the three largest barriers that men faced upon their release, with stability aspects, such as positive relationships, religion, and support groups, being key facilitators to their successful reintegration. The implications for future research, and policy and practice, including prioritizing risk assessment and management, offering appropriate and timely treatment and rehabilitation, educating the community, better access to housing and employment, and services adopting a collaborative approach, are discussed.

## Background

Rates of sexual offending recidivism are reported to be lower than that of general offenders (Justice Inspectorates, 2021). Although this can be partially accounted for by the level of risk management directed to people convicted of sexual offenses and that recidivism rates are often underestimated since sexual offenses are rarely reported or unlikely to be resolved by arrest (Przybylski, 2015), individuals who have committed a sexual offense are conceptualized as more dangerous than other offenders (Sample & Bray 2003). While such conceptualizations may be warranted due to the costs sexual offenses incur to society, (e.g., increased fear of crime, psychological or physical impact on the primary victims [Resilience, 2018], anger, and financial costs [Loya, 2015]), this renders reintegration difficult for those who have committed a sexual offense.

This presents a significant problem when considering the volume of ex-offenders needing support with reintegration. In 2021 there were 95,844 registered individuals who had committed a sex offense in England and Wales (Full Fact, 2021), and a total of 917,771 in the United States of America (USA) (Vigderman & Turner, 2021). Those who have been or are about to be released from prison will potentially need treatment, interventions, mental health support, accommodation, and employment to reduce the risk of recidivism and promote safe community reintegration.

Several protective factors contribute to the cessation of sexual offending, including supportive relationships (Kras, 2019), access to pro-social activities, employment opportunities, suitable and safe housing, access to education and treatment, and participation in offender interventions (Harris et al., 2017). Previous research has also found that familial relationships and social support have greater power over human behavior than sanctions, restrictions, and punishments, with the latter often negatively impacting community reintegration and encouraging reoffending (Cooley et al., 2017).

The restrictions and punishments unique to those convicted of sexual offenses are primarily a result of community public notification systems, such as “Megan’s Law” in the USA, which informs the community when a person convicted of sexual offenses moves into their neighborhood, and “Sarah’s Law” in the U.K., which allows anyone to formally ask the police if someone who has access to a child has a child sexual offense record. This could be considered an extreme form of punishment that no other group of offenders faces (Corrigan, 2006). It is therefore recommended that more support is provided for men who have been convicted of sexual offenses (MCoSO) when reintegrating back into the community, which could include resilience building, less severe formal social control, and more therapeutic services (Kras, 2022).

These findings demonstrate the need for improvement across the criminal justice system, and overall, there is a need to fully identify and explore what can help or hinder the reintegration process for MCoSO to reduce reoffending rates and make recommendations to support MCoSO through this process that is based on evidence and the needs of this offender group. This is particularly important when considering the high number of sexual offenses committed across the world and the impact this crime has upon individuals and society as a whole. Therefore, this qualitative systematic review aims to identify the full extent of the barriers and facilitators that MCoSO face when reintegrating back into the community.

## Methods

To address the above aim, a systematic review was conducted on qualitative-only studies in order to gather in-depth, rich, and insightful understandings of the barriers and facilitators MCoSO faces when reintegrating back into the community.

A systematic review was chosen over a meta-synthesis to present the findings in a structured manner, consistent with the approach taken by other studies (Campbell et al., 2020). Systematic reviews offer a rigorous and transparent method for synthesizing a large body of qualitative research, facilitating a comprehensive understanding of a particular phenomenon. Through explicit and transparent methods, systematic reviews minimize bias in the selection, appraisal, and synthesis of qualitative studies. Additionally, they inform policy and practice decisions by identifying key themes and synthesizing available evidence (Shaheen et al., 2023). The protocol for this systematic review was registered with PROSPERO (CRD42023409254), with one amendment being made since its registration (this amendment included three changes: replaced Endnote with Rayyan, added a team member, and amended to look at the views from MCoSO only). The review is reported in line with Preferred Reporting Items for Systematic Reviews and Meta-Analyses 2020 reporting guidelines (Shamseer et al., 2015).

### Search Strategy

Fourteen electronic databases were searched: Psych Info, MEDLINE, Psych Articles, Psychology and Behavioral Sciences Collection, Web of Science, ASSIA, Scopus, Criminal Justice Abstracts, CINAHL, Taylor & Francis Online, Science Direct, SAGE Journals, PubMed, and ProQuest, and two gray literature databases were searched; MEDNAR and Google Scholar, with the first 100 hits being retrieved. One researcher (ET) ran the searches from the 16 databases.

Searches of all databases were conducted in line with the PEO (Population, Exposure, and Outcomes) framework (Mattisson, 2023) for conducting literature searches for qualitative synthesis. The PEO framework is useful for qualitative-only reviews (McEwan, 2023) and can help determine the association between particular exposures/risk factors and outcomes which can help to inform policy and practice (Munn et al., 2018). Elements of the PEO framework were used to inform the keywords and identify relevant papers. Population included men convicted of a sexual offense, Exposure included reintegration, and Outcome included barriers and facilitators. An example search strategy can be found in Supplemental Material File 1. All searches were conducted in April 2023, and to ensure all relevant papers were captured, gold standard papers (those that matched the research questions) were searched for and found to ensure the sensitivity of the search.

### Eligibility Criteria

Papers were included if they used qualitative research methods about the barriers, facilitators, perceptions, experiences, and attitudes toward community reintegration from prison or secure care for MCoSO. Papers were included if other offense types were present, such as homicide or drug offenses, if the data on MCoSO could be extracted. Similarly, papers were included that did not specifically focus on reintegration if data on reintegration were presented and could be extracted. Additionally, for the papers to be eligible, they needed to include the experiences and perspectives of adult (over the age of 18) males convicted of a sexual offense and could be from any country and any year.

### Study Selection and Data Management

All results from the database search were imported into Rayyan for storage, duplication detection, and sifting. One reviewer (ET) sifted all titles and abstracts against the inclusion criteria. The results were sent to a second reviewer (NC) who independently double-screened 10%. Any discrepancies were discussed between reviewers, and if an agreement could not be reached one reviewer would make a final decision (MC). There was a 97.87% agreement rate between reviewers (substantial agreement rate when converted into Kappa statistic). Discrepancies did not go to a third reviewer. Following titles and abstract sifting, the papers identified as potentially relevant went through the second sifting phase of full paper screening. All full texts were identified, retrieved, and saved on Microsoft Teams for review. One reviewer (ET) sifted all full papers, and a second reviewer (MC) independently double-screened 20%. There was a 100% agreement rate between reviewers (perfect agreement rate when converted into Kappa statistic).

### Data Extraction

A Microsoft Excel spreadsheet (the Microsoft Corporation, Redmond, Washington) was developed for the data extraction, which captured the authors, year of publication, country of study, the aim of the research, study design, methods, setting/location of research, sample size, prison/community based, participant demographics, included barriers, included facilitators, and recommendations. One reviewer (ET) undertook the data extraction, and another reviewer (MC) checked 20% of the papers against the extracted data to ensure data was not missing or there were no errors.

### Assessment of Quality

The critical appraisal skills programme (CASP) tool for appraisal of qualitative studies (Singh, 2013) was used for the quality assessment of the included papers and was undertaken by one reviewer (ET). Another reviewer (NC) checked 20% of the papers and quality scores with no discrepancies found. There was a 100% agreement rate between reviewers (perfect agreement rate when converted into Kappa statistic). The CASP tool was chosen as it has been endorsed by Cochrane and the World Health Organization for use in qualitative evidence synthesis and is a relatively good measure of the transparency of research practice and reporting standards (Long et al., 2020). No papers were excluded based on the quality score.

### Synthesis

Thematic synthesis was utilized in the findings of the included studies. Thematic synthesis was chosen as it offers a systematic and rigorous approach to synthesizing qualitative data and allows flexibility, transparency, and the ability to generate new insights, making it a valuable methodological choice. Additionally, thematic synthesis involves identifying and organizing key themes or patterns within a large volume of data, which helps to develop a comprehensive understanding of the research topic (Thomas & Harden, 2008). Verbatim quotations from MCoSO either inside prison/secure care or in the community were extracted from the papers, and NVivo 10 (Lumivero, Denver, Colorado) was used to complete the free line-by-line coding, which was then constructed into descriptive themes and finally developed into analytical themes. ET completed initial drafts of the synthesis, and then the wider study team agreed upon the themes and sub-themes, which allowed a broader view of the experiences and perceptions of reintegrating into the community for those who had been convicted of a sexual offense and enhanced the trustworthiness and rigor of the synthesis.

## Results

**Figure 1.**
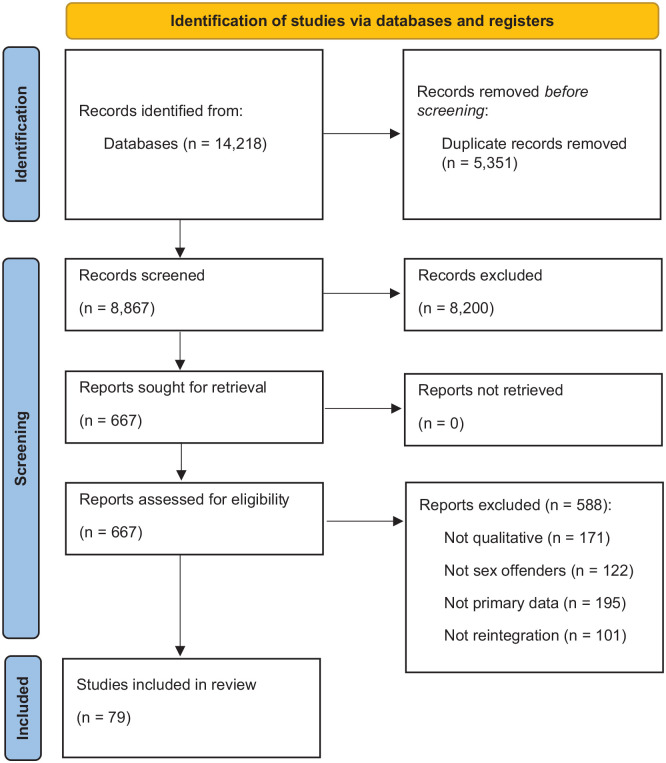
Preferred reporting items for systematic review and meta-analysis.

The initial searches yielded 14,218 records. After de-duplication (*n* = 5,351 total removed) and title and abstract sifting (*n* = 8,200 removed), 667 full papers were assessed. In total, 79 papers (11 of which re-used the same sample across studies) met the inclusion criteria and were included in the review (Figure 1). The articles were published between 2000 and 2023.

### Study Characteristics

The characteristics from the 79 qualitative papers are presented in the Supplemental Material. All the papers included male participants, with an average age of 45 (ranging from 18 to 82). The majority of offenders were white (78%) and had a minimum of a high school diploma (45.7%). Almost half of the men were single (49.3%) and had employment (47.7%), which earned them $30,000 or less (65.4%). Fourteen papers focused on the perspectives of reintegration of men inside prison, sixty-two papers explored the experiences of reintegration of men in the community, two papers looked at men in both prison and the community, and one looked at the perspectives of reintegration from men in secure care. In total, 3,527 unique MCoSO were included as participants. Seventy papers included a mixture of sexual offense types; six specifically looked at child sex offenses, one focused only on adult rapists, one paper explored offenses against children and vulnerable adults, and lastly, one paper specifically focused on image-based internet sexual offenses. Forty-six papers reported on research from the United States of America, 22 from the United Kingdom, 3 from the Netherlands, 3 from Australia and Canada, and one each from New Zealand, Italy, and Norway.

### Thematic Results

Six emerging themes were found, which surrounded three main areas: formal aspects (registration, notification, and probation, and interventions, therapy, and support groups), stability aspects (accommodation, employment and education, and relationships and religion), and cultural and societal barriers (labeling, stigma, and vigilantism), which this section will now explore further. Grouping these themes together ensured comprehensive coverage of the various factors related to the reintegration of MCoSO into society. By examining formal aspects, stability aspects, and cultural and societal barriers, the systematic review provided a holistic understanding of the challenges and opportunities for reintegration.

#### Theme 1: Registration, Notification, and Probation

Registration, notification, and probation were found to have caused shame, stigma, and isolation and to be a strain in the reentry process, with common feelings of injustice. Forty-seven papers focused on this area and first found that many of the participants who were in prison lacked knowledge of the registration laws and did not feel prepared for this upon their release: “I don’t know what the rules are, I’ve heard different things, I’ll have to check it out when the time comes” (Tewksbury & Copes, 2013, p. 107).

Many of the participants in the studies believed that sex offender registration and notification were useless and not a deterrent for reoffending (which is its main purpose); instead, it was seen as an unnecessary and prolonged additional condition put upon the men to further punish them (Cooley et al., 2017; Kemshall et al., 2012; Murphy & Fedoroff, 2013; Seidler, 2010; Tewksbury & Lees, 2007). The length of registration was also seen as a barrier as many men were lifelong registrants and therefore felt they had nothing to work toward or see any hope in their futures (Collins et al., 2010; Cooley et al., 2017; Griffin & Evans, 2021; Harris & Levenson, 2022; Kitson-Boyce et al., 2019a; Mann et al., 2021; Tewksbury & Connor, 2012; Tovey et al., 2022): “I feel as though I am being doubly punished. . .I can never stop registering. I want to leave America, but felons can’t. I can’t wait for the lord to take me from this hell” (Ackerman et al., 2013, p. 38). Additionally, the consequences of the sex offender registry and notification appeared monumental for the men and included thoughts of suicide, hopelessness, anger, being harassed and discriminated against, causing isolation, and the loss of careers, positive futures, and friends and family (Griffin & Evans, 2021; Harris & Levenson, 2022; Kras, 2022; Levenson & Cotter, 2005b; Levenson et al., 2007; Seidler, 2010; Tewksbury & Lees, 2007; Tewksbury, 2013; Zevitz & Farkas, 2000):

I am dealing with a lot of anger, distrust, and hopelessness. My goals for the American dream are shattered. Why set those goals for myself when everything I would hope to accomplish can easily be taken away from me . . . I have lost all hope that I will ever have a productive and enjoyable life because of this registration (Ackerman et al., 2013, p. 37).

Others expressed how unfair they felt it was altogether and that they had to register the same as other MCoSO who may have committed “worse crimes” than them and argued for it to be distinguishable as they did not want to be categorized in this way (Ackerman et al., 2013; Digard, 2014; Evans & Cubellis, 2015; Kras, 2022; Levenson et al., 2007; Lytle et al., 2017; Murphy & Fedoroff, 2013; Seidler, 2010):

They don’t differentiate between the guy that goes out and goes to a party and runs across a 16-year-old girl and has oral sex with her or the guy that drags a 5-year-old off the playground and rapes and kills her. It’s still a sex offender (Tewksbury & Lees, 2007, p. 396).

On the other hand, some of the men viewed the registry as a way to help keep them on track with their lives, with some offering recommendations on how to make the process better, for example being able to differentiate between MCoSO on the registry and having a clinician to review registrants regarding their readiness to come off the list (Cooley et al., 2017; Ievins & Mjåland, 2021; Levenson et al., 2007; Murphy & Fedoroff, 2013; Seidler, 2010; ten Bensel & Sample, 2017):

I believe the registry is a good thing for some people, but there are a few people that the registry is not really helping them or the public . . . Each case should be individually analyzed to see if that person should even be placed on the registry (Ackerman et al., 2013, p. 39).

Ahead of living with the stigma of the sex offender label, many participants stated that parole and probation restrictions were the most flawed part of the reintegration process and hurt their daily lives. Twelve papers explored this further and discovered other reasons for this, including the lack of support that was given to the men (before and after release), how judgmental probation officers were of them, the lack of trust between them, and generally how inflexible they were toward them (Ackerman & Brown, 2020; Allan et al., 2023; Bailey & Sample, 2017; Cooley et al., 2017; Digard, 2014; Ievins & Mjåland, 2021; Mann et al., 2021; Russell et al., 2013; Seidler, 2010; Simmons et al., 2022; Zevitz & Farkas, 2000): “Probation doesn’t help. Their job is to re-arrest you because they are told you are a threat to society and you’re going to reoffend. You’re a time bomb. It’s just a matter of time” (McCartan et al., 2021, p. 1306). In addition to these feelings, many of the men felt worried, especially when thinking anything could trigger them being recalled to prison. Their interactions with probation services did not reduce those feelings as they described feeling they were being constantly monitored (Burchfield & Mingus, 2008; Harris & Levenson, 2021; Ievins & Mjåland, 2021; Kitson-Boyce et al., 2018; ten Bensel & Sample, 2019). However, some of the men felt that going back to prison would be a better option than remaining on probation: “All this indefinite stuff is just so draining. There’s nothing to hope for. . . it can even get to a stage where, well, actually going to prison isn’t so bad” (Tovey et al., 2022, p. 16).

Restrictions and conditions imposed by the probation service hugely impacted the men’s lives, especially surrounding what they could do with their families, where they could live, and the specific problems arising from polygraph testing and electronic monitoring (Digard, 2014). In regards to spending time with their families and interacting socially with others, many barriers stood in the men’s way as a result of their restrictions (Dubois & Ouellet, 2020; Harris et al., 2019; Liem & Weggemans, 2018; Paat et al., 2017; Zevitz & Farkas, 2000).

When it came to residency restrictions, the men struggled to accept their conditions due to the complexities and the unpredictable nature of the world around them, which often led to confusion as to why these conditions were there in the first place: “The reality is that people—including children—are everywhere, not just restricted areas” (Page et al., 2012, p. 125). Electronic monitoring was also seen as an unnecessary and highly burdensome condition placed upon the men, which often led to them losing their jobs and confidence, as well as missing out on activities they used to enjoy doing, such as swimming, yoga, and dancing. Many viewed the device as a “ball and chain” which reminded them they were “bad” and did not see it as a deterrent to reoffend but just another way to punish them after they have been released (Burchfield & Mingus, 2008; Juventa & Krim, 2017; Kemshall et al., 2012; Youssef et al., 2023).

Polygraphs were equally seen as a waste of resources that did not affect reoffending. There was skepticism about the polygraph’s accuracy, with many believing it was inefficient:

It is an extremely negative tool to use to sort of say to somebody we don’t believe that you’re following your license conditions . . . but it assumes such a negative thing to sort of say we don’t believe you until the machine tells us (Spruin et al., 2018, p. 21).

Lastly, when examining the responses from the very few who expressed positivity about probation, it indicated how much of a difference it makes to the men’s lives when they encounter understanding and supportive staff who appeared to care about the men in a professional manner (Cooley et al., 2017).

She’s, she’s very, very knowledgeable about sex offenders and sex offending. And never hesitated to share with me how inappropriate she thinks that some of the laws are. And at the same time, never came close to excusing sex offending either (Bailey & Sample, 2017, p. 195).

#### Theme 2: Interventions, Therapy, and Support Groups

Individuals convicted of sexual offenses are often required to undergo therapy or attend intervention-based meetings either inside prison and/or once released into the community. The purpose of this is to educate and help the ex-prisoners to build an offense-free life (Youssef et al., 2023). Seven papers explored men’s experiences and feelings toward this and found there was a mixture of responses from the men regarding interventions and therapy. Some found many benefits to attending these courses/therapy once being released, whereas others did not understand the need for them and viewed it as an unnecessary requirement that they had already undertaken in prison, therefore rendering it a “waste of time” (Allan et al., 2023; Robbers, 2009; Youssef et al., 2023). However, it is important to note that it is one thing going through prison treatment, where dealing with temptation is hypothetical, and doing it in a community where the temptations are real.

However, many men described how useful it had been for them (Dervley et al., 2017; Hollomotz, 2021; Kras, 2022; Robbers, 2009; Youssef et al., 2023). This included benefits to the men’s relationships, being able to help others, and giving them a positive outlook on their future: “When you do something of this nature you feel you’re a pariah to society. . .and the easiest way of solving the problem is to opt out of society. The course took that away. It gave me a future” (Dervley et al., 2017, p. 53). Looking specifically at support groups, there are a limited number of them that welcome men with sexual convictions in the community, and for those that do, there is little awareness of them. Support groups can be extremely beneficial and helpful to men when they are reintegrating into the community as they are a place where they can be open, honest, and build friendships (Sample et al., 2022). More established support groups, such as Circles of Support and Accountability (CoSA), have been successful in helping released men convicted of sexual offenses reenter society positively while also encouraging them to take accountability for what they have done. Eighteen papers focused on CoSA and examined the facilitators surrounding the support they give to the men. CoSA was often compared to probation services, whereby it was discovered that CoSA provided the men with a more positive environment that was free of judgment, and it was suggested that other recent MCoSO are made aware of their services (Bohmert et al., 2018; McCartan et al., 2021): “You could be more open with a Circle rather than Probation Officer or when you are in prison, I could talk about risk and then would decide among themselves if it was cumulative” (Ackerman & Brown, 2020, p. 26). It also appeared CoSA gave the men much more support than statutory provisions could offer in different areas of their lives, including helping them look for jobs, providing advice, strengthening family relationships, and allowing friendships to form (Fox, 2015, 2016, 2017; Furse & Kitson-Boyce, 2023; Höing et al., 2013, 2017; Kitson-Boyce et al., 2019b).

Additionally, the peer-support group Fearless (based in the USA) provided men a place to meet with others who also had sexual convictions to offer advice and reduce isolation (Sample et al., 2018, 2022): “I don’t know what I would do without those guys. They show me that they have survived and so can I. I really think my membership in Fearless helped get me through my lonely times” (ten Bensel & Sample, 2019, p. 286). The men felt less specialized support groups were unfamiliar with potential sources of help for MCoSO. Several papers centered around this and the lack of awareness, availability, and benefits of these groups. Firstly, the lack of awareness and availability of support was apparent in many of the papers, which revealed the need for the existence and promotion of such groups: “I had no groups, not aware of anything—for example, Samaritans. I wasn’t aware of anything. As a sex offender I’m concerned what’s available. I need to know what’s available. I would take any support I could. I questioned if it applied to me” (Ackerman & Brown, 2020, p. 24). The men believed support groups not only helped them to reintegrate into society but also reduced their chances of reoffending (Lytle et al., 2017; Simmons et al., 2022; Zevitz & Farkas, 2000): “Once I talked, I felt better. . .. Just knowing others struggle and win helps me to remember I can too” (ten Bensel & Sample, 2017, p. 506).

#### Theme 3: Accommodation

Sixteen papers discussed the barriers MCoSO faced when trying to find and secure accommodation, with only one paper reporting a positive experience. “Yes, my landlord knows about it because I had to disclose to him and he was very good about it, he has been brilliant about it” (Kemshall et al., 2012, p. 320). Most men, when released from prison for a sexual offense, experience considerable difficulty finding and retaining appropriate housing that abides by their specific probation conditions (Bohmert et al., 2018; Harris & Levenson, 2021; Page et al., 2012; Russell et al., 2013). The difficulties in finding accommodation following release from prison reportedly stemmed from the lack of support, information, and guidance given to the men by probation and other services (Allan et al., 2023; Russell et al., 2013): “I was forced to live on the streets for a while because probation services could not find a house that was suitable for someone like me” (Liem & Weggemans, 2018, p. 483). Another common difficulty was the reluctance of landlords and communities to have a “sex offender” living in their area (Burchfield & Mingus, 2008; Cresswell, 2020; Harris & Levenson, 2021; Rydberg, 2018; Zevitz & Farkas, 2000).

The accommodation available for the men (often hostels, halfway houses, shelters, and transitional facilities), were described as being “like a prison,” “awful,” “costly,” “chaotic,” and “warehouses for sex offenders” and as places they could not escape from (Ackerman & Brown, 2020; Dubois & Ouellet, 2020; Harris et al., 2019; Kras et al., 2016; Rydberg, 2018; Tewksbury, 2012). This led to one man stating that he re-offended to escape his environment. “I had no support in a parole hostel. I wanted to go back to my home area, so I offended to get back to that area” (Ackerman & Brown, 2020, p. 26).

#### Theme 4: Employment and Education

Twenty-seven papers discussed the effects of being unable to find employment, as well as what having a job meant to MCoSO. Finding a job with a criminal record is a major challenge, and even more so with a sexual offense (Dubois & Ouellet, 2020). Commonly, MCoSO have limited career options and are often prevented from returning to their former employment (Dubois & Ouellet, 2020; Harris et al., 2014; Mann et al., 2021; McAlinden et al., 2017; Rydberg, 2018; Sandbukt, 2021; Schaefer et al., 2004; Simmons et al., 2022).

Most men who were interviewed in the papers had lost their jobs following conviction and were unemployed but looking for work. Reasons given for unemployment included fears of disclosure, being recognized, being asked questions about their offense at interview, threats, and harassment because they were on the sex offender register: “I want to work but I just back down, because they ask if you have any criminal history—“can we run a record check on you?” and I’m like, “forget it, forget it’” (Harris et al., 2014, p. 1572). This was also not helped by the lack of perceived support from probation services upon their release regarding advice on finding employment and about the lengthy conditions they had to abide by so as not to return to prison (Faccio et al., 2020; Liem & Weggemans, 2018; Mann et al., 2021; Rydberg, 2018; Schaefer et al., 2004; Zevitz & Farkas, 2000): “I think they could have helped me with jobs that are prone to ex-cons, the info provided is out of date” (Allan et al., 2023, p. 68).

Another barrier was the attitudes of employers, which in some cases led to men lying about their offense type or about having a criminal record to secure employment (Mann et al., 2021; Robbers, 2009). Most employers, when finding out about their previous convictions, would not consider hiring the men, with many not getting to the end of their interviews. This led to men either not gaining employment or accepting jobs that were below their skill levels: “I had to tell them in a previous interview for a job. . . their faces just fall. It’s ok I understand. . . it’s being punished more than the sentence in a way” (Mann et al., 2021, p. 216).

Overall, employment was a large focus of the papers, as acquiring a job once released from prison was the men’s top priority as gave them hope for the future (Allan et al., 2023; Farmer et al., 2012; Harris et al., 2014; McAlinden et al., 2017; Russell et al., 2013; Rydberg, 2018; Schaefer et al., 2004; Tewksbury & Copes, 2013; Tovey et al., 2022; Youssef et al., 2023). Employment was also seen as a facilitator to reintegrating and helped the men desist from reoffending (Faccio et al., 2020, p. 129).

Three papers discussed education, which appeared crucial to some of the men once they had returned to the community as they were able to focus on other things in their life and aim for a better future, as well as helping them to acquire a job: “Absolutely! I think that education is critical. I would advise everybody to go back and get more education, yeah. And that is the key to changing the whole mindset” (Tewksbury, 2013, p. 18). However, barriers were common surrounding the educational environment, including stigmatization (Harris et al., 2019; Slater et al., 2023). In addition, due to fear of exposure, campus registrants reported having few social interactions and friends and a very limited range of activities beyond the classroom:

You have the registry as well as your campus registry. It’s isolating, and it can make it more difficult to communicate to people. I myself am feeling cut off from people, which is probably one of the more dangerous positions to be in for somebody who is an RSO (Tewksbury, 2013, p. 9).

#### Theme 5: Relationships and Religion

Relationships, whether that be with friends, family, or spouses, were often seen as either detrimental or crucial to the men’s success in reintegrating into the community. Thirty-six papers explored MCoSO relationships and how this helped or hindered their reintegration process. Positive relationships helped the men in all areas of their life, such as gaining employment, having a place to live, building their confidence, and feeling supported and less isolated: “My parents been (sic) helping me out financially . . . support, advice, just about anything my parents can do to help me out, they have been. I actually feel like my family wants me around” (Kras, 2019, p. 41). However, many participants reported that their sexual conviction had ruined their relationships with family, friends, and spouses, which led to the men feeling abandoned, lonely, and segregated from society: “I got emptiness. Now I’ve lost my boys and it’s kinda’ torn everybody apart” (Schaefer et al., 2004, p. 232). While others felt their status of being a “sex offender” was impacting their relationships and stopped them from being able to enjoy their life as they did beforehand with their loved ones (Davis Frenzel et al., 2014; Dubois & Ouellet, 2020; Ievins & Mjåland, 2021; Lytle et al., 2017; Mann et al., 2021). Men reported being fearful and reluctant to peruse or maintain relationships due to their “sex offender” label:

I’m afraid of doing anything. This whole experience for me has been very traumatic in the sense it’s completely changed the way I do things, pretty much the way I live. For the past 2 years I’ve pretty much secluded myself. I mean I don’t even go visit my family (Harris & Levenson, 2022, p. 9).

Most participants in the seven studies examining religion felt their relationship with religion, and God was important and indicated that they felt safe within their churches and were less fearful of being ostracized among churchgoers than the general community: “Church and Christ forgives, the law and society doesn’t” (Kewley et al., 2017, p. 90). Religion was also viewed as a facilitator of reintegration, with some of the men pledging to be more committed to the church once they were released (Allan et al., 2023): “Buddhism will have a big influence on the way I live from now on” (Bell et al., 2018, p. 6). However, it was evident that the men faced barriers to attending church because of their sexual convictions, for example, needing prior permission from pastors to attend services or requirement of having to be accompanied by another adult (Harris et al., 2017, 2019):

#### Theme 6: Labeling, Stigma, and Vigilantism

Thirty-eight papers discussed labeling and stigma, which was often found to lead to suicidal thoughts, ostracism, discrimination, assaults on character and reputation, prejudgments of character, malicious rumors, intrusions of privacy, community petitions, and threats to property and person:

I thought of suicide because I felt people were talking bad about me. Some people want for me to die. That’s what this law is about, to cause enough stress on the offender so he will take his own life (Levenson & Cotter, 2005a, p. 59).

Firstly, looking at perceptions, prison-confined MCoSC spoke of what they thought it would be like to reintegrate into the community. The fear and knowledge of the stigmatization of MCoSO started early in the prison sentence, with many anticipating negative reactions from society: “You’re a sex offender, you ain’t nothing now. You got all of them against you . . . That’s the way people think out there. Sex offender? Garbage! Shouldn’t even come back out on the street, we’re not allowed out there” (Tewksbury & Copes, 2013, p. 111). Community residing MCoSO experienced stigmatization and felt their conviction defined who they were and how they will always be remembered, which also led to embarrassment for their family members: “I know it’s gotta be difficult for him [brother] just to have the same last name. . .guilty by association. He’s carrying part of the stigma that I do just because he carries the same last name” (Burchfield & Mingus, 2008, p. 365). The men lived in constant fear of somebody recognizing them and lacked control over who knew about their crime and the anticipated reprisals (Burchfield & Mingus, 2008; Cresswell, 2020; Fox, 2015; Harris & Levenson, 2021; Robbers, 2009; Sandbukt, 2021; Tovey et al., 2022): “It’s just a horrible feeling knowing that it’s out there and it’s public information. And, everybody and anybody can get into it. I hate it” (Tewksbury & Lees, 2006, p. 330).

A lot of the men could not understand why only sexual crimes are highly stigmatized, and others are not (e.g., murderers and burglars). Additionally, the men reported feeling that society, including the criminal justice system, perceived all “sex offenders” as equally reprehensible and potentially violent and dangerous:

We are like dirt . . . We made a mistake. A robber made a mistake. A murderer made a mistake. They’re all free to go when they get out of here. Me? When I leave out of here, I’m tagged for the rest of my life (Tewksbury & Copes, 2013, p. 111).

The men also spoke about how having a “sex offender” label affects most areas of their lives, including job opportunities, going to the shop, rehabilitation, speaking to others, not being regarded as a citizen, limited opportunities, and harsher consequences (Dubois & Ouellet, 2020; Harris & Levenson, 2021; Harris et al., 2014; Ievins & Mjåland, 2021; Liem & Weggemans, 2018; Russell et al., 2013; Sandbukt, 2021; Slater et al., 2023; ten Bensel & Sample, 2019; Tewksbury & Lees, 2006; Tewksbury & Copes, 2013; Worley & Worley, 2013).

In some cases, stigmatization and labeling contribute to instances of vigilantism, where individuals or groups take it upon themselves to seek retribution against those convicted of a sexual offense outside the legal system. Vigilante actions can range from public shaming and harassment to physical violence (Cubellis et al., 2019). Seven papers discussed the men’s fear and experiences of vigilantism. Repercussions of the “sex offender” label and notification laws left men worried for their safety due to the actions of vigilantes. This led to men being motivated to reoffend out of anger and fear (Murphy & Fedoroff, 2013; Woodall et al., 2013). Men’s fears were fueled by both the stories of others and the media portrayals of MCoSO (Burchfield & Mingus, 2008; Harris & Levenson, 2021; Liem & Weggemans, 2018; Zevitz & Farkas, 2000). Additionally, some of the men had been targeted by vigilante groups (Burchfield & Mingus, 2008; Worley & Worley, 2013; Zevitz & Farkas, 2000).

## Discussion

This qualitative systematic review highlighted six emerging themes that revolved around the challenges that MCoSO faced during the reintegration process. These themes encompassed formal aspects, stability aspects, and cultural and societal barriers, with each shedding light on the varied nature of the obstacles encountered by MCoSO, providing valuable insights for policymakers, practitioners, and academics. It appeared that these barriers and facilitators were not specific to certain countries but were similar across all regions of the world; however, there were some distinct differences, specifically for the American research participants. It was apparent that American laws and communities were more punitive toward MCoSO, evidenced in the residency restrictions (for example, not being permitted to live near schools or playgrounds) and public notifications, which in some cases were found to lead to vigilantism.

### Barriers to Reintegration

The discussion surrounding parole and probation revealed a flawed and inflexible system that added to the challenges of reintegration. Participants described probation officers as judgmental and unsupportive, exacerbating feelings of constant surveillance and fear of re-arrest. While the restrictions placed on MCoSO, such as the prohibition of internet access, are intended to safeguard society, there is a growing recognition that these measures can have unintended consequences, impacting the lives of individuals who may not pose a significant risk (Chan et al., 2016). Denying internet access, a fundamental tool in today’s interconnected world, can disproportionately affect their ability to reintegrate into society, including finding employment, whereby job searches, applications, and networking are predominantly conducted online. Many aspects of our daily routines have transitioned to the online realm, encompassing activities such as scheduling appointments with general practitioners and paying bills. The inability to utilize online resources not only hinders their job-seeking efforts but also restricts their capacity to acquire necessary skills and knowledge (Tewksbury & Zgoba, 2010). The restriction, when imposed on individuals who may not present a high risk of reoffending, raises concerns about fairness and the potential hindrance to rehabilitation efforts (Hutt, 2019).

The duration of registration also emerged as a significant barrier, with lifelong registration contributing to a sense of hopelessness and a lack of motivation for positive change. Moreover, the consequences of “sex offender registration” were profound, extending beyond legal implications to encompass emotional and psychological distress. The perceived unfairness of treating all MCoSO uniformly, regardless of the severity of their offenses, further fueled discontent. These findings are consistent with previous quantitative studies, which reiterate the damage these extra conditions create (Eddleman, 2022), the perceived lack of support the men receive in this area, and the negative experiences the registry and probation services cause (Wolf, 2021). Interestingly, while some individuals saw the registry as a potential tool for maintaining accountability and facilitating reintegration, a considerable number advocated for a fairer approach, which echoed the recommendations proposed by current research. Suggestions included individualized analysis for registry placement, elimination of residence restrictions, and involvement of clinicians in assessing readiness for removal from the list (Levenson, 2018).

Gaining accommodation and employment were viewed as highly important factors and crucial to the success of community reintegration (Baker et al., 2021) since they enabled MCoSOs to aim for better futures. MCoSO’s often face challenges in the realm of employment, frequently finding themselves relegated to under-skilled work despite their prior experience in more specialized fields (Grossi, 2017; Harris et al., 2020). Many may have held skilled positions prior to prosecution, and it is crucial to recognize the importance of aligning job opportunities with their existing skill sets (Wooldridge & Bailey, 2023). For instance, a former teacher might still possess the ability to teach but could redirect their expertise toward a context like instructing prisoners rather than children. Beyond the vocational aspect, the reintegration process demands a concerted effort to challenge their mindset and provide a sense of purpose; therefore, it is essential to offer employment opportunities that motivate and engage, acknowledging the profound link between one’s identity and their occupation. For many men, their job is not just a means of financial stability; it profoundly shapes their identity, impacting self-esteem, social standing, and relationships (Longhi et al., 2023). Societal expectations and traditional gender roles further reinforce the link between a man’s identity and his professional achievements, highlighting the crucial role of meaningful work in contributing to well-being and a sense of purpose (Dicke et al., 2019).

The final barrier explored the issues of labeling, stigma, and the threat of vigilantism faced by MCoSO, which were described as the most damaging to reintegration (in relation to physical and mental health). Participants expressed anticipation of negative reactions, both from society at large and within their communities, reinforcing the sense of being permanently marked by their offenses. The fear of vigilantism was palpable among MCoSO, with concerns about safety and instances of targeted harassment by vigilante groups. This fear, in some cases, contributed to a cycle of anger and desperation, prompting individuals to contemplate reoffending as a means of escape (Cubellis et al., 2019). Similar research aiming to reduce barriers to reintegration for a largely stigmatized population has found that by fostering open and informed discussions, communities can gain a better understanding of the complexities surrounding negatively labeled individuals (Wessells, 2006). Through community mobilization, education campaigns can dispel myths, emphasizing the importance of rehabilitation and reintegration into society, with community leaders, including law enforcement and local organizations, playing a pivotal role in promoting empathy and discouraging vigilantism. Ultimately, by encouraging a more compassionate and rehabilitative approach, communities can work collaboratively to build safer environments while respecting the rights and dignity of all individuals involved.

### Facilitators to Reintegration

Unique to this review, the findings indicated a mixed response among individuals regarding the efficacy of mandated interventions and therapy. While some viewed these programs as beneficial, providing an opportunity for personal growth and positive changes, others perceived them as redundant, particularly if they had already undergone similar programs in prison. However, the importance of support groups, especially those like CoSA, was evident in facilitating positive reintegration. CoSA, in contrast to traditional probation services, provided a supportive and non-judgmental environment that addressed various aspects of the individuals’ lives, including employment, family relationships, and community integration. There has been a considerable amount of literature concerning CoSA and its effectiveness in helping MCoSO successfully reintegrate into the community and provide additional support to the men and their spouses (Clarke et al., 2017). This has been achieved by the volunteer circle members offering emotional support and understanding, emphasizing accountability, rebuilding social skills, and providing education and resources.

CoSA, initially rooted in religious foundations (McCartan et al., 2014), is undergoing a resurgence by incorporating faith elements, which have recently been introduced in New Zealand (Lowe & Willis, 2019). However, the impact of this shift remains uncertain and requires thorough evaluation. As part of this evolving approach, increasing religious services and involving more faith leaders could be crucial to the men’s reintegration. As highlighted in this review, religion, fundamentally centered on forgiveness, holds the potential to play a significant role in the rehabilitation and reintegration of MCoSO into society. Emphasizing this core value within faith communities may foster understanding, compassion, and support for individuals seeking to rebuild their lives after release.

Although largely under-resourced, CoSA and other charity organizations, such as the Lucy Faithful Foundation, are recognized for providing support to MCoSO and fostering positive relationships with their family and friends, as well as safeguarding children (Bailey et al., 2018; Richards, 2021). Consistent with current research, this review found that positive relationships were greatly valued by MCoSO as they reportedly helped them to reintegrate into the community and rebuild their lives by building confidence, escaping judgment, and helping to gain employment (Harris & Levenson, 2022). These relationships are also found to be crucial in preventing reoffending and suicide attempts (Absalom, 2021; Walker et al., 2020). However, it is important to note the often-overlooked impact the crimes have on family members of those convicted of this particular offense. The revelation of such offenses can lead to significant emotional turmoil, shame, and social isolation for the family while also having to contend with societal judgment, financial loss, and having to navigate and engage with the criminal justice system (Armitage et al., 2023). Recognizing the need for support services for the family members of MCoSO, CoSA aims to address this emotional impact by providing a safe space for sharing experiences and guiding family members in coping with the challenges they face (Wager et al., 2015).

Overall, it is apparent that more work is required to help MCoSO successfully reintegrate into the community and start their journey to becoming productive members of society, significantly reducing the chances of recidivism. This includes providing MCoSO support in helping them find suitable housing, employment, and education, as well as offering appropriate treatment, interventions, and/or therapy, along with careful considerations over license conditions. Therefore, policies and practices in MCoSO reintegration should be evidence-based [as opposed to policies based upon community pressure and fear (Kernsmith et al., 2016)] and be continuously evaluated and informed by research. Collecting data on reoffending rates, treatment outcomes, and the effectiveness of various reintegration strategies can help identify areas for improvement and guide evidence-based policy development.

### Study Limitations

This systematic review revealed that across different types of MCoSO worldwide, similar barriers emerged when examining community reintegration. This finding provides reassurance that the challenges faced by MCoSO in reintegrating into society are consistent across various contexts. By identifying and synthesizing these common barriers, this review contributes to a more generalizable understanding of factors affecting community reintegration for MCoSO, thereby facilitating the development of more effective interventions and support programs; however, several limitations need to be considered. It is important to note that this review is primarily based on subjective data, using people subjected to criminal sanctions to discuss how fair and effective these sanctions are, and, therefore, may be compelled to minimize, justify, or shift blame. Additionally, the research environment in the studies may have had honesty and openness compromised due to the limits of confidentiality that often arise when working with offenders. Another limitation surrounds the inability to disaggregate the data based on the types of sexual offenses that lead to the convictions and the problem of presenting the findings from across different jurisdictions and countries where the policies and practices differ markedly. Additionally, this review only included qualitative studies, therefore, it is recommended a mixed-methods or quantitative review be undertaken in this area. It is also important to acknowledge the limitations of using a systematic review approach. Systematic reviews may oversimplify the complexities of qualitative research by reducing findings to common themes and patterns. Additionally, they may be susceptible to publication bias if they only include published studies. Lastly, it is important to consider the diversity of the participant samples. All the participants were male, most were white, and many came from the USA; therefore, the findings may not be generalizable to other races, cultures, or females convicted of a sexual offense.

## Conclusion

To the best of the authors’ knowledge, this is the first systematic review of studies reporting on the barriers and facilitators to MCoSO community reintegration. This qualitative systematic review highlights that to overcome the barriers in MCoSO reintegrating back into the community and promoting the facilitators, a comprehensive and multi-dimensional approach is required that addresses various factors. These include providing specialized therapy, counseling, and interventions tailored to MCoSO-specific needs, creating supportive and structured environments that facilitate positive change, assisting MCoSO in obtaining meaningful employment and vocational training, ensuring access to stable and suitable housing, rebuilding positive relationships with family and friends, raising public awareness and educating the community about sex offenses, rehabilitation, and reintegration, and continual evaluation and improvement of legal and policy frameworks. By implementing these strategies, society can support MCoSO in their efforts to reintegrate into the community, further reduce the risk of reoffending, and promote a safer and more inclusive society. This requires a balance between public safety concerns and the recognition of individuals’ potential for rehabilitation and successful community reintegration.

**Table 1.** Critical Findings.

Firstly, we found that formal and cultural aspects of reintegration, such as probation services, stigmatization, and registration, were the three largest barriers that men faced upon their release into the community. These barriers also caused some men’s mental health to decline or worsen after their release.
Secondly, we found stability aspects, such as positive relationships, religion, and support groups, to be key facilitators for men who have been convicted of sexual offenses (MCoSOs) successful reintegration, helping them to settle into the community much easier and efficiently.
Thirdly, we found that overall, MCoSO lacked knowledge while inside prison about what it would be like once they were released and generally felt unprepared to reintegrate back into the community.

**Table 2.** Implications for Policy and Practice.

Sex offender assessments need to be conducted in consultation with the men who have been convicted of sexual offenses (MCoSO) so that they can understand why they are categorized at particular risk levels. This can help inform decisions regarding supervision, treatment, and community notification and offer the chance to remove the offender from certain restrictions when risk is no longer apparent.
Sex offender treatment programs and therapy: An explanation needs to be given to MCoSO as to why they are being asked to complete a program for a second time (once inside and next outside of prison).
Policies should focus on educating the public about sex offenses, their causes, and effective strategies for prevention and safeguarding. Community members should be provided with accurate information and guidance to dispel misconceptions, reduce the stigma associated with MCoSO, and enhance their capacity to protect themselves and others from undetected sexual predators. Open dialog and public awareness campaigns can help foster a supportive environment for the reintegration of MCoSO.
Policies should address the challenges faced by MCoSO in finding stable housing and employment. This may involve working with housing authorities and employers to develop fair policies and practices that consider the individual’s risk level, progress in treatment, and other relevant factors.
Effective MCoSO reintegration requires collaboration among various stakeholders, including law enforcement, prisons, treatment providers, community organizations, and other relevant services. Policies should facilitate information sharing, coordination, and collaboration among these stakeholders to ensure a comprehensive approach to reintegration.

## Supplemental Material

sj-docx-1-tva-10.1177_15248380241254080 – Supplemental material for A Qualitative Systematic Review of the Barriers and Facilitators of the Reintegration of Men Convicted of a Sexual Offense From Prison or Secure Care into the Community Supplemental material, sj-docx-1-tva-10.1177_15248380241254080 for A Qualitative Systematic Review of the Barriers and Facilitators of the Reintegration of Men Convicted of a Sexual Offense From Prison or Secure Care into the Community by Emma Tuschick, Nikki Carthy, Nadia Wager and Marty Chamberlain in Trauma, Violence, & Abuse

sj-docx-2-tva-10.1177_15248380241254080 – Supplemental material for A Qualitative Systematic Review of the Barriers and Facilitators of the Reintegration of Men Convicted of a Sexual Offense From Prison or Secure Care into the Community Supplemental material, sj-docx-2-tva-10.1177_15248380241254080 for A Qualitative Systematic Review of the Barriers and Facilitators of the Reintegration of Men Convicted of a Sexual Offense From Prison or Secure Care into the Community by Emma Tuschick, Nikki Carthy, Nadia Wager and Marty Chamberlain in Trauma, Violence, & Abuse

## References

[bibr1-15248380241254080] Ackerman A. R. Sacks M. Osier L. N. (2013). The experiences of registered sex offenders with internet offender registries in three states. Journal of Offender Rehabilitation, 52(1), 29–45. 10.1080/10509674.2012.720959

[bibr2-15248380241254080] Ackerman G. Brown C. (2020). “No one told me about Circles”: Perspectives of circles of support and accountability and perceived support to prevent sexual reoffending. Prison Service Journal, 1(251).

[bibr3-15248380241254080] Allan C. Winters G. M. Grydehøj R. F. Jeglic E. L. Calkins C. (2023). Perspectives of reentry and desistance: A comparison of individuals convicted of general and sexual offenses. Journal of Offender Rehabilitation, 62(1), 59–80. 10.1080/10509674.2022.2158983

[bibr4-15248380241254080] Absalom L. (2021). Indecent images of children offending and suicide: An interpretative phenomenological analysis of partners’ perspectives [Doctoral dissertation, UCL (University College London)].

[bibr5-15248380241254080] Armitage R. Wager N. Wibberley D. Hudspith L. F. Gall V. (2023). “We’re not allowed to have experienced trauma. We’re not allowed to go through the grieving process”-Exploring the indirect harms associated with Child Sexual Abuse Material (CSAM) offending and its impacts on non-offending family members. Victims & Offenders, 1,1–27.

[bibr6-15248380241254080] Bailey A. Squire T. Thornhill L. (2018). The Lucy Faithfull Foundation: Twenty-five years of child protection and preventing child sexual abuse. In Sexual crime and prevention (pp. 57–82). Springer Link, Rebecca Lievesley.

[bibr7-15248380241254080] Bailey D. J. S. Sample L. L. (2017). Sex offender supervision in context. Criminal Justice Policy Review, 28(2), 176–204. 10.1177/0887403415572876

[bibr8-15248380241254080] Baker T. Zgoba K. Gordon J. A. (2021). Incarcerated for a sex offense: In-prison experiences and concerns about reentry. Sexual Abuse, 33(2), 135–156. 31679468 10.1177/1079063219884588

[bibr9-15248380241254080] Bell K. Winder B. Blagden N. (2018). “Better as a Buddhist”: An interpretative phenomenological analysis of the reflections on the religious beliefs of buddhist men serving a prison sentence for a sexual offense. Religions, 9(4), 101. 10.3390/rel9040101

[bibr10-15248380241254080] Bohmert M. N. Duwe G. Hipple N. K. (2018). Evaluating restorative justice circles of support and accountability: Can social support overcome structural barriers? International Journal of Offender Therapy and Comparative Criminology, 62(3), 739–758. 10.1177/0306624X16652627 27272526

[bibr11-15248380241254080] Burchfield K. B. Mingus W. (2008). Not in my neighborhood. Criminal Justice and Behavior, 35(3), 356–374. 10.1177/0093854807311375

[bibr12-15248380241254080] Campbell F. Booth A. Hackett S. Sutton A. (2020). Young people who display harmful sexual behaviors and their families: A qualitative systematic review of their experiences of professional interventions. Trauma, Violence, & Abuse, 21(3), 456–469. 10.1177/1524838018770414 29739282

[bibr13-15248380241254080] Chan E. J. McNiel D. E. Binder R. L. (2016). Sex offenders in the digital age. Journal of the American Academy of Psychiatry and the Law Online, 44(3), 368–375. 27644871

[bibr14-15248380241254080] Clarke M. Brown S. Völlm B. (2017). Circles of support and accountability for sex offenders: A systematic review of outcomes. Sexual Abuse, 29(5), 446–478. 26369806 10.1177/1079063215603691

[bibr15-15248380241254080] Collins E. Brown J. Lennings C. (2010). Qualitative review of community treatment with sex offenders: Perspective of the offender and the expert. Psychiatry, Psychology, and Law, 17(2), 290–303. 10.1080/13218710903421282

[bibr16-15248380241254080] Cooley B. N. Moore S. E. Sample L. L. (2017). The role of formal social control mechanisms in deterring sex offending as part of the desistance process. Criminal Justice Studies, 30(2), 136–157. 10.1080/1478601X.2017.1299335

[bibr17-15248380241254080] Corrigan R. (2006). Making meaning of Megan’s Law. Law & Social Inquiry, 31(2), 267–312. 10.1111/j.1747-4469.2006.00012.x

[bibr18-15248380241254080] Cresswell C. (2020). “Why would you choose to study sex offenders?”: Assisted desistance and reintegration of perpetrators of sexual harm. Irish Probation Journal, 17, 63–86.

[bibr19-15248380241254080] Cubellis M. A. Evans D. N. Fera A. G. (2019). Sex offender stigma: An exploration of vigilantism against sex offenders. Deviant Behavior, 40(2), 225–239.

[bibr20-15248380241254080] Davis Frenzel E. Bowen K. N. Spraitz J. D. Bowers J. H. Phaneuf S . (2014). Understanding collateral consequences of registry laws: An examination of the perceptions of sex offender registrants. Justice Policy Journal, 11(2), 1–22.

[bibr21-15248380241254080] Dervley R. Perkins D. Whitehead H. Bailey A. Gillespie S. Squire T. (2017). Themes in participant feedback on a risk reduction programme for child sexual exploitation material offenders. Informa UK Limited.

[bibr22-15248380241254080] Dicke A. L. Safavian N. Eccles J. S. (2019). Traditional gender role beliefs and career attainment in STEM: A gendered story?. Frontiers in Psychology, 10, 1053. 10.3389/fpsyg.2019.01053 PMC6519300 31139116

[bibr23-15248380241254080] Digard L. (2014). Encoding risk: Probation work and sex offenders’ narrative identities. Punishment & Society, 16(4), 428–447. 10.1177/1462474514539536

[bibr24-15248380241254080] Dubois M. Ouellet F. (2020). Les défis de la réinsertion sociale. Criminologie (Montréal), 53(2), 309–334. 10.7202/1074197ar

[bibr25-15248380241254080] Eddleman T. L. (2022). Social priming: Registered sex offenders’ perceptions of sex offender registration and notification. New Jersey City University.

[bibr26-15248380241254080] Evans D. N. Cubellis M. A. (2015). Coping with stigma: How registered sex offenders manage their public identities. American Journal of Criminal Justice, 40(3), 593–619. 10.1007/s12103-014-9277-z

[bibr27-15248380241254080] Faccio E. Mazzucato M. Iudici A. (2020). Discursive chains: How prison becomes real and chains identity movements for a sex offender. International Journal for Crime, Justice and Social Democracy, 9(4), 118–134. 10.5204/ijcjsd.v9i2.1434

[bibr28-15248380241254080] Farmer M. Beech A. R. Ward T. (2012). Assessing desistance in child molesters. Journal of Interpersonal Violence, 27(5), 930–950. 10.1177/0886260511423255 22203639

[bibr29-15248380241254080] Fox K. J. (2015). Theorizing community integration as desistance-promotion. Criminal Justice and Behavior, 42(1), 82–94. 10.1177/0093854814550028

[bibr30-15248380241254080] Fox K. J. (2016). Civic commitment: Promoting desistance through community integration. Punishment & Society, 18(1), 68–94. 10.1177/1462474515623102

[bibr31-15248380241254080] Fox K. J. (2017). Contextualizing the policy and pragmatics of reintegrating sex offenders. Sexual Abuse, 29(1), 28–50. 10.1177/1079063215574711 25792536

[bibr32-15248380241254080] Full Fact. (2021). There are far fewer than 850,000 registered sex offenders in the UK. https://fullfact.org/online/sex-offenders-registry/

[bibr33-15248380241254080] Furse G. Kitson-Boyce R. (2023). “Why moan about it?” An IPA analysis of ex-core members’ experience of a pandemic without a CoSA. SAGE. 10.1177/0306624X231159879 PMC10014443 36912264

[bibr34-15248380241254080] Griffin V. W. Evans M. (2021). The duality of stigmatization: An examination of differences in collateral consequences for Black and White sex offenders. Justice Quarterly, 38(6), 1019–1046. 10.1080/07418825.2019.1666906

[bibr35-15248380241254080] Grossi L. M. (2017). Sexual offenders, violent offenders, and community reentry: Challenges and treatment considerations. Aggression and Violent Behavior, 34, 59–67.

[bibr36-15248380241254080] Harris D. A. Ackerman A. Haley M. (2014). Desistance from sexual offending: Findings from 21 life history narratives. Criminal Justice Studies, 30(2), 101–116. 10.1080/1478601X.2017.1300385 24424253

[bibr37-15248380241254080] Harris D. A. Ackerman A. Haley M. (2017). “Losing my religion:” an exploration of religion and spirituality in men who claim to have desisted from sexual offending. Criminal Justice Studies, 30(2), 101–116. 10.1080/1478601X.2017.1300385

[bibr38-15248380241254080] Harris D. A. Levenson J. (2021). Life on “the list” is a life lived in fear: Post-conviction traumatic stress in men convicted of sexual offenses. International Journal of Offender Therapy and Comparative Criminology, 65(6–7), 763–789. 10.1177/0306624X20952397 32851869

[bibr39-15248380241254080] Harris D. A. Levenson J. S. (2022). A framework for post-conviction traumatic stress: Preliminary findings from a focus group of men under community supervision for sex offenses. Informa UK Limited.

[bibr40-15248380241254080] Harris D. A. Pedneault A. Willis G. (2019). The pursuit of primary human goods in men desisting from sexual offending. Sexual Abuse, 31(2), 197–219. 10.1177/1079063217729155 28874094

[bibr41-15248380241254080] Harris M. Tynan R. Kimmett D. (2020). Thinking Differently Employers’ views on hiring people convicted of sexual offences. https://unlock.org.uk/wp-content/uploads/misc/Thinking-Differently-PRT-Unlock-report.pdf

[bibr42-15248380241254080] Höing M. Bogaerts S. Vogelvang B. (2013). Circles of support and accountability: How and why they work for sex offenders. Journal of Forensic Psychology Practice, 13(4), 267–295. 10.1080/15228932.2013.808526

[bibr43-15248380241254080] Höing M. A. Vogelvang B. Bogaerts S. (2017). I am a different man now’ Sex offenders in Circles of Support and Accountability: A prospective study. International Journal of Offender Therapy and Comparative Criminology, 61(7), 751–772. 10.1177/0306624X15612431 26510629

[bibr44-15248380241254080] Hollomotz A. (2021). Successful community resettlement of men with learning disabilities who have completed a hospital-based treatment for sexual offending. The British Journal of Social Work, 51(1), 150–169. 10.1093/bjsw/bcaa038

[bibr45-15248380241254080] Hutt J. (2019). Offline: Challenging internet and social media bans for individuals on supervision for sex offenses. New York University Review of Law & Social Change, 43, 663.

[bibr46-15248380241254080] Ievins A. Mjåland K. (2021). Authoritarian exclusion and laissez-faire inclusion: Comparing the punishment of men convicted of sex offenses in England & Wales and Norway*. Wiley.

[bibr47-15248380241254080] Justice Inspectorates. (2021). Sexual offending. https://www.justiceinspectorates.gov.uk/hmiprobation/research/the-evidence-base-probation/specific-sub-groups/sexual-offending/

[bibr48-15248380241254080] Juventa B. Krim (2017). “Stalked by the state”: GPS surveillance technology and sex offender parolees. Kriminologisches Journal, 49(2), 103–119.

[bibr49-15248380241254080] Kemshall H. Dominey J. Hilder S. (2012). Public disclosure: Sex offenders’ perceptions of the pilot scheme in England. Compliance, legitimacy and living a Good Life. The Journal of Sexual Aggression, 18(3), 311–324. 10.1080/13552600.2011.603062

[bibr50-15248380241254080] Kernsmith P. Comartin E. Kernsmith R. (2016). Fear and misinformation as predictors of support for sex offender management policies. Journal of Sociology & Social Welfare, 43, 39.

[bibr51-15248380241254080] Kewley S. Larkin M. Harkins L. Beech A. R. (2017). Restoring identity: The use of religion as a mechanism to transition between an identity of sexual offending to a non-offending identity. Criminology & Criminal Justice, 17(1), 79–96. 10.1177/1748895816654530

[bibr52-15248380241254080] Kitson-Boyce R. Blagden N. Winder B. Dillon G. (2018). A prison-model of CoSA: The potential to offer through the gate support and accountability. The Journal of Sexual Aggression, 24(3), 294–310. 10.1080/13552600.2018.1509575

[bibr53-15248380241254080] Kitson-Boyce R. Blagden N. Winder B. Dillon G. (2019a). “This time it’s different” Preparing for release through a prison-model of CoSA: A phenomenological and repertory grid analysis. Sexual Abuse, 31(8), 886–907. 10.1177/1079063218775969 29790431

[bibr54-15248380241254080] Kitson-Boyce R. Blagden N. Winder B. Dillon G. (2019b). Supporting desistance through ambiguous practice: What can be learned from the first prison-based model of CoSA in England and Wales? Journal of Forensic Psychology Research and Practice, 19(2), 186–209. 10.1080/24732850.2019.1571362

[bibr55-15248380241254080] Kras K. R. (2019). Can social support overcome the individual and structural challenges of being a sex offender? Assessing the social support-recidivism link. International Journal of Offender Therapy and Comparative Criminology, 63(1), 32–54. 10.1177/0306624X18784191 29947562

[bibr56-15248380241254080] Kras K. R. (2022). The irredeemable? How men convicted of sexual offenses reflect and reconcile redemption and condemnation scripts on the path to desistance. Deviant Behavior, 43(11), 1293–1312. 10.1080/01639625.2021.1977592

[bibr57-15248380241254080] Kras K. R. Pleggenkuhle B. Huebner B. M. (2016). A new way of doing time on the outside. International Journal of Offender Therapy and Comparative Criminology, 60(5), 512–534. 10.1177/0306624X14554194 25326464

[bibr58-15248380241254080] Levenson J. S. (2018). Sex offender management policies and evidence-based recommendations for registry reform. Current Psychiatry Reports, 20, 1–7. 29560559 10.1007/s11920-018-0884-0

[bibr59-15248380241254080] Levenson J. S. Cotter L. P. (2005a). The effect of Megan’s Law on sex offender reintegration. Journal of Contemporary Criminal Justice, 21(1), 49–66. 10.1177/1043986204271676

[bibr60-15248380241254080] Levenson J. S. Cotter L. P. (2005b). The impact of sex offender residence restrictions: 1,000 feet from danger or one step from absurd? International Journal of Offender Therapy and Comparative Criminology, 49(2), 168–178. 10.1177/0306624X04271304 15746268

[bibr61-15248380241254080] Levenson J. S. D Amora D. A. Hern A. L. (2007). Megan’s law and its impact on community re-entry for sex offenders. Behavioral Sciences & the Law, 25(4), 587–602. 10.1002/bsl.770 17620324

[bibr62-15248380241254080] Liem M. Weggemans D. (2018). Reintegration among high-profile ex-offenders. Journal of Developmental and Life-Course Criminology, 4(4), 473–490. 10.1007/s40865-018-0093-x 30873339 PMC6390714

[bibr63-15248380241254080] Long H. A. French D. P. Brooks J. M. (2020). Optimising the value of the critical appraisal skills programme (CASP) tool for quality appraisal in qualitative evidence synthesis. Research Methods in Medicine & Health Sciences, 1(1), 31–42. 10.1177/2632084320947559

[bibr64-15248380241254080] Longhi S. Nandi A. Bryan M. Connolly S. Gedikli C. (2023). Life satisfaction and unemployment—The role of gender attitudes and work identity. Scottish Journal of Political Economy, 71, 219–236.

[bibr65-15248380241254080] Lowe G. Willis G. (2019). Looking inside a circle: Volunteer experiences of circles of support and accountability. Psychiatry, Psychology and Law, 26(1), 129–149. 10.1080/13218719.2018.1485521 PMC6762178 31984069

[bibr66-15248380241254080] Loya R. M. (2015). Rape as an economic crime: The impact of sexual violence on survivors’ employment and economic well-being. Journal of Interpersonal Violence, 30(16), 2793–2813. 25381269 10.1177/0886260514554291

[bibr67-15248380241254080] Lytle R. Bailey D. J. S. ten Bensel T. (2017). We fought tooth and toenail: Exploring the dynamics of romantic relationships among sex offenders who have desisted. Criminal Justice Studies, 30(2), 117–135. 10.1080/1478601X.2017.1299322

[bibr68-15248380241254080] Mann N. Devendran P. N. Lundrigan S. (2021). “You’re never really free”: Understanding the barriers to desistance for registered sexual offenders in the community. Criminology & Criminal Justice, 21(2), 206–223. 10.1177/1748895819853861

[bibr69-15248380241254080] Mattisson R. (2023). LibGuides: Search, evaluate, write and cite: Framing a Research Question. https://libguides.lub.lu.se/c.php?g=666801&p=4840125

[bibr70-15248380241254080] McAlinden A. Farmer M. Maruna S. (2017). Desistance from sexual offending: Do the mainstream theories apply? Criminology & Criminal Justice, 17(3), 266–283. 10.1177/1748895816670201

[bibr71-15248380241254080] McCartan K. F. Harris D. A. Prescott D. S. (2021). Seen and not heard: The service user’s experience through the justice system of individuals convicted of sexual offenses. International Journal of Offender Therapy and Comparative Criminology, 65(12), 1299–1315. 10.1177/0306624X19851671 31132910

[bibr72-15248380241254080] McCartan K. Kemshall H. Westwood S. Solle J. MacKenzie G. Cattel J. Pollard A. (2014). Circles of Support and Accountability (CoSA): A case file review of two pilots. London, United-Kingdom: The University of West England, De Montfort University and the Ministry of Justice.

[bibr73-15248380241254080] McEwan A. (2023). LibGuides: Forming answerable search questions using frameworks: PEO. Libguides.exeter.ac.uk. https://libguides.exeter.ac.uk/c.php?g=655921&p=5039312

[bibr74-15248380241254080] Munn Z. Stern C. Aromataris E. Lockwood C. Jordan Z. (2018). What kind of systematic review should I conduct? A proposed typology and guidance for systematic reviewers in the medical and health sciences. BMC Medical Research Methodology, 18(1), 1–9. 29316881 10.1186/s12874-017-0468-4 PMC5761190

[bibr75-15248380241254080] Murphy L. Fedoroff J. P. (2013). Sexual offenders’ views of Canadian sex offender registries: A survey of a clinical sample. Canadian Journal of Behavioural Science/Revue canadienne des sciences du comportement, 45(3), 238. 10.1037/a0033251

[bibr76-15248380241254080] Paat Y. Hope T. L. Lopez L. C. Zamora H. Salas C. M. (2017). Hispanic ex convicts’ perceptions of challenges and reintegration. Journal of Offender Rehabilitation, 56(2), 87–109. 10.1080/10509674.2016.1268233

[bibr77-15248380241254080] Page A. D. Hill J. S. Gilbert G. (2012). North Carolina sexual offender legislation: Policy llacebo? Journal of Offender Rehabilitation, 51(3), 115–134. 10.1080/10509674.2011.623219

[bibr78-15248380241254080] Page M. J. McKenzie J. E. Bossuyt P. M. Boutron I. Hoffmann T. C. Mulrow C. D. Shamseer L. Tetzlaff J. M. Akl E. A. Brennan S. E. Chou R. (2021). The PRISMA 2020 statement: An updated guideline for reporting systematic reviews. BMJ, 372, n71. 10.1136/bmj.n71 PMC8005924 33782057

[bibr79-15248380241254080] Przybylski R. (2015). Chapter 5: Adult sex offender recidivism. Office of Sex Offender Sentencing, Monitoring, Apprehending, Registering, and Tracking. https://smart.ojp.gov/somapi/chapter-5-adult-sex-offender-recidivism

[bibr80-15248380241254080] Resilience. (2018). Effects of sexual violence. https://www.ourresilience.org/what-you-need-to-know/effects-of-sexual-violence/

[bibr81-15248380241254080] Richards K. (2021). Desistance from sexual offending: The role of circles of support and accountability. Routledge.

[bibr82-15248380241254080] Robbers M. L. P. (2009). Lifers on the outside. International Journal of Offender Therapy and Comparative Criminology, 53(1), 5–28. 10.1177/0306624X07312953 18268079

[bibr83-15248380241254080] Russell G. Seymour F. Lambie I. (2013). Community reintegration of sex offenders of children in New Zealand. International Journal of Offender Therapy and Comparative Criminology, 57(1), 55–70. 10.1177/0306624X11426132 22100427

[bibr84-15248380241254080] Rydberg J. (2018). Employment and housing challenges experienced by sex offenders during reentry on parole. Corrections: Policy, Practice and Research, 3(1), 15–37. 10.1080/23774657.2017.1369373

[bibr85-15248380241254080] Sample L. L. Bray T. M. (2003). Are sex offenders dangerous?. Criminology & Public Policy, 3(1), 59–82.

[bibr86-15248380241254080] Sample L. L. Cooley B. N. ten Bensel T. (2018). Beyond circles of support: “Fearless”—An open peer-to-peer mutual support group for sex offense registrants and their family members. International Journal of Offender Therapy and Comparative Criminology, 62(13), 4257–4277. 10.1177/0306624X18758895 29478390

[bibr87-15248380241254080] Sample L. L. Cooley B. N. Garman J. D. (2022). “Fearless” revisited: How this peer-to-peer self-help group for sex offense registrants and their families continued to operate during a global pandemic. SAGE. 10.1177/0306624X221132217 36326269

[bibr88-15248380241254080] Sandbukt I. J. (2021). Reentry in practice: Sexual offending, self-narratives, and the implications of stigma in Norway. SAGE. 10.1177/0306624X211049184 34605278

[bibr89-15248380241254080] Schaefer B. M. Friedlander M. L. Blustein D. L. Maruna S. (2004). The work lives of child molesters. Journal of Counseling Psychology, 51(2), 226–239. 10.1037/0022-0167.51.2.226

[bibr90-15248380241254080] Seidler K. (2010). Community management of sex offenders: Stigma versus support. Sexual Abuse in Australia and New Zealand, 2(2), 66–76. http://search.informit.org/doi/10.3316/informit.052210567079497

[bibr91-15248380241254080] Shaheen N. Shaheen A. Ramadan A. Hefnawy M. T. Ramadan A. Ibrahim I. A. Hassanein M. E. Ashour M. E. Flouty O. (2023). Appraising systematic reviews: A comprehensive guide to ensuring validity and reliability. Frontiers in Research Metrics and Analytics, 8, 1268045. 38179256 10.3389/frma.2023.1268045 PMC10764628

[bibr92-15248380241254080] Shamseer L. Moher D. Clarke M. Ghersi D. Liberati A. Petticrew M. Shekelle P. Stewart L. A. (2015). Preferred reporting items for systematic review and meta-analysis protocols (PRISMA-P) 2015: Elaboration and explanation. BMJ, 350, g7647. 10.1136/bmj.g7647 25555855

[bibr93-15248380241254080] Simmons M. Kim B. Hyde J. Lemon T. L. Scharer K. E. McInnes D. K. (2022). Protecting the public’s health through successful reentry for sex offender after incarceration. Journal of Interpersonal Violence, 37(17–18), NP15231–NP15254. 10.1177/08862605211016344 PMC8617060 34039087

[bibr94-15248380241254080] Singh J. (2013). Critical appraisal skills programme. Journal of Pharmacology & Pharmacotherapeutics, 4(1), 76–77. 10.4103/0976-500X.107697

[bibr95-15248380241254080] Slater J. Winder B. Grady A. Banyard P. (2023). “There is nothing for me”: A qualitative analysis of the views towards prison education of adult male prisoners convicted of a sexual offense. Wiley.

[bibr96-15248380241254080] Spruin E. Wood J. L. Gannon T. A. Tyler N. (2018). Sexual offender’s experiences of polygraph testing: A thematic study in three probation trusts. The Journal of Sexual Aggression, 24(1), 12–24. 10.1080/13552600.2017.1378025

[bibr97-15248380241254080] ten Bensel T. Sample L. L . (2017). The influence of sex offender registration and notification laws on fostering collective identity among offenders. Journal of Crime & Justice, 40(4), 497–511. 10.1080/0735648X.2015.1131184

[bibr98-15248380241254080] ten Bensel T. Sample L. L . (2019). Social inclusion despite exclusionary sex offense laws: How registered citizens cope with loneliness. Criminal Justice Policy Review, 30(2), 274–292. 10.1177/0887403416675018

[bibr99-15248380241254080] Tewksbury R. (2012). Stigmatization of sex offenders. Deviant Behavior, 33(8), 606–623. 10.1080/01639625.2011.636690

[bibr100-15248380241254080] Tewksbury R. (2013). Sex offenders and campus-based sex offender registration: Stigma, vulnerability, isolation, and the classroom as refuge. Journal of Qualitative Criminal Justice & Criminology, 1(2), 221–242. 10.21428/88de04a1.e537a5f0

[bibr101-15248380241254080] Tewksbury R. Connor D. P. (2012). Incarcerated sex offenders’ perceptions of family relationships: Previous experiences and future expectations. Criminology, Criminal Justice, Law & Society, 13(2), 25.

[bibr102-15248380241254080] Tewksbury R. Copes H. (2013). Incarcerated sex offenders’ expectations for reentry. The Prison Journal (Philadelphia, PA), 93(1), 102–122. 10.1177/0032885512467318

[bibr103-15248380241254080] Tewksbury R. Lees M. (2006). Perceptions of sex offender registration: Collateral consequences and community experiences. Sociological Spectrum, 26(3), 309–334. 10.1080/02732170500524246

[bibr104-15248380241254080] Tewksbury R. Lees M. B. (2007). Perceptions of punishment. Crime and Delinquency, 53(3), 380–407. 10.1177/0011128706286915

[bibr105-15248380241254080] Tewksbury R. Zgoba K. M. (2010). Perceptions and coping with punishment: How registered sex offenders respond to stress, internet restrictions, and the collateral consequences of registration. International Journal of Offender Therapy and Comparative Criminology, 54(4), 537–551. 19561135 10.1177/0306624X09339180

[bibr106-15248380241254080] Thomas J. Harden A. (2008). Methods for the thematic synthesis of qualitative research in systematic reviews. BMC Medical Research Methodology, 8(1), 45. 10.1186/1471-2288-8-45 PMC2478656 18616818

[bibr107-15248380241254080] Tovey L. Winder B. Blagden N. (2022). “It’s ok if you were in for robbery or murder, but sex offending, that’s a no no”: A qualitative analysis of the experiences of 12 men with sexual convictions seeking employment. Psychology, Crime & Law, 29(6), 653–676. 10.1080/1068316X.2022.2030736

[bibr108-15248380241254080] Vigderman A. Turner G. (2021). United States cities ranked by the frequency of registered sex offenders. Security.org. https://www.security.org/blog/u-s-cities-ranked-by-the-frequency-of-registered-sex-offenders/

[bibr109-15248380241254080] Wager N. M. Wager A. R. Wilson C. (2015). Circles South East’s programme for non-offending partners of child sex offenders: A preliminary outcome evaluation. Probation Journal, 62(4), 357–373.

[bibr110-15248380241254080] Walker A. Kazemian L. Lussier P. Na C. (2020). The role of family support in the explanation of patterns of desistance among individuals convicted of a sexual offense. Journal of Interpersonal Violence, 35(17–18), 3643–3665. 29294774 10.1177/0886260517712273

[bibr111-15248380241254080] Wolf B. (2021). Barriers to sex offender reintegration [Doctoral dissertation, Walden University].

[bibr112-15248380241254080] Woodall J. Dixey R. South J. (2013). Prisoners’ perspectives on the transition from the prison to the community: Implications for settings-based health promotion. Critical Public Health, 23(2), 188–200. 10.1080/09581596.2012.732219

[bibr113-15248380241254080] Wooldridge J. L. Bailey D. J. (2023). “I’m Not Unemployed, I’m Unemployable”: Challenges finding and sustaining work for people required to register as sex offenders. qualitativecriminology.com

[bibr114-15248380241254080] Worley R. M. Worley V. B. (2013). The sex offender next door: Deconstructing the United States’ obsession with sex offender registries in an age of neoliberalism. International Review of Law, Computers & Technology, 27(3), 335–344. 10.1080/13600869.2013.796708

[bibr115-15248380241254080] Youssef C. Casey S. Birgden A. Guadagno B. (2023). The significance of an Australian community maintenance program for men who have sexually offended—Participant perspectives. Journal of Forensic Psychology Research and Practice, 23(1), 56–91. 10.1080/24732850.2021.2013365

[bibr116-15248380241254080] Zevitz R. G. Farkas M. A. (2000). From the psychiatric hospital to the community: Integrating conditional release and contingency management. Behavioral Sciences & the Law, 18(2–3), 375. 10.1002/1099-079 11018777

