# Supplementary material for: A Qualitative Systematic Review of the Barriers and Facilitators of the Reintegration of Men Convicted of a Sexual Offense From Prison or Secure Care into the Community
Source: Trauma Violence Abuse. 2024 May 28;25(5):3615–30. doi: 10.1177/15248380241254080 (PMC11545130; doi:10.1177/15248380241254080)
Supplement: sj-docx-1-tva-10.1177_15248380241254080 – Supplemental material for A Qualitative Systematic Review of the Barriers and Facilitators of the Reintegration of Men Convicted of a Sexual Offense From Prison or Secure Care into the Community [file sj-docx-1-tva-10.1177_15248380241254080.docx]

**Supplementary File 1. Search Terms**

| **P**opulation | Sexual offenders | “sexual offenders” OR “sex offenders” OR “rapists” OR “child sexual abusers” OR “paedophiles” OR “pedophiles” OR “child molester” OR “sex abuser” OR “sexual abuser” OR “online predator” OR “internet-facilitated sexual offenders” OR “CSEM offenders” OR “incest offender” |
| --- | --- | --- |
| **E**xposure | Reintegration | “reintegration” OR “re-entry” OR “reentry” OR “community” OR “transition” OR “resettlement”  “employment” OR “job” OR “work” OR “housing” OR “accommodation” OR “approved premises” OR “settlement” OR “education” OR “training” OR “community notification” OR “registry” OR “disclosure” OR “conditions” |
| **O**utcome | Barriers and Facilitators | “barriers” OR “facilitators” OR “attitudes” OR “experiences” OR “perceptions” OR “stigma*” OR “emotions” OR “risk” OR challeng*” OR “fear” OR “obstacle” OR “negative” OR “positive” OR “knowledge” OR “confidence” OR “understand” OR “help” OR “motivation” OR “awareness” OR “apprehension” OR “beliefs” OR “factors” OR “opinions” OR “problem” OR “thoughts” OR “restrictions” or “support” OR “mental health” OR “mental illness” |
