# Supplementary material for: A Qualitative Systematic Review of the Barriers and Facilitators of the Reintegration of Men Convicted of a Sexual Offense From Prison or Secure Care into the Community
Source: Trauma Violence Abuse. 2024 May 28;25(5):3615–30. doi: 10.1177/15248380241254080 (PMC11545130; doi:10.1177/15248380241254080)
Supplement: sj-docx-2-tva-10.1177_15248380241254080 – Supplemental material for A Qualitative Systematic Review of the Barriers and Facilitators of the Reintegration of Men Convicted of a Sexual Offense From Prison or Secure Care into the Community [file sj-docx-2-tva-10.1177_15248380241254080.docx]

**Supplementary File 2. Study Characteristics**

| **Author, Year, Context, Location** | **Methods** | **Sample size** | **Age** | **Victim Age/ offence type** | **Themes included** |
| --- | --- | --- | --- | --- | --- |
| Ackerman et al., 2013, community, USA | Survey | 80 | Adult | Mixture | 1 |
| Akerman & Brown, 2020, prison, UK | Focus Group | 3 | Adult | Adults | 1, 2, 3, 5, 6 |
| Allan et al., 2023, community, USA | Interviews & survey | 106 | Adult | Mixture | 1 - 6 |
| Bailey & Sample, 2017, community, USA | Interviews | 8 | Mean 56. | Mixture | 1 |
| Bell et al., 2018, prison, UK | Interviews | 7 | Mean 42 | Mixture | 5 |
| Bohmert et al., 2018, community, USA | Interviews | 10 | Mean 38 range 25-51 | Mixture | 2, 3, 5 |
| Burchfield & Mingus, 2008, community, USA | Interviews | 23 | < 25 (8.7%), 25-64 (87%), 65> (4.3%). | Mixture | 1, 3, 4, 5, 6 |
| Collins et al., 2010, community, Australia | Interviews | 8 | Range 43-74 | Mixture | 1 |
| Connor & Copes, 2011, prison, USA | Interviews | 24 | Mean age: 40.6 | Mixture | 2 |
| Cooley et al., 2017, community, USA | Interviews | 77 | Mean 40, range 25-82 | Mixture | 1 |
| Cresswell, 2020, community, UK | Interviews & survey | 18 | Adult | Mixture | 3, 4, 5, 6 |
| Davis Frenzel et al., 2014, community, USA | Survey | 443 | Mean 50.3 | Mixture | 4, 5, 6 |
| Dervley et al., 2017, community, UK | Interviews | 13 | Mean 47.3 | Internet | 2 |
| Digard, 2014, community, USA | Interviews | 18 | Range 25-75 | Mixture | 1, 6 |
| Dubois & Ouellet, 2020, community, Canada | Interviews | 16 | Range 24-68 | Mixture | 1, 3, 4, 5, 6 |
| Evans & Cubellis, 2015, community, USA | Interviews | 20 | Median 37 | Mixture | 1, 5, 6 |
| Faccio et al., 2020, prison, Italy | Interviews & survey | 32 | Adult | Mixture | 4, 6 |
| Farmer et al., 2012, community, UK | Interviews | 10 | Mean 41 | Mixture | 4, 5 |
| Fox, 2015, community USA | Interviews & focus groups | 20 | Adult | Mixture | 2, 6 |
| Fox, 2016, community, USA | Interviews & focus groups | 20 | Adult | Mixture | 2 |
| Fox, 2017, community, USA | Interviews | 28 | Adult | Mixture | 2 |
| Furse & Kitson-Boyce, 2023, community, UK | Interviews | 4 | Range 36-79 | Mixture | 2, 5 |
| Griffin & Evans, 2021, community, USA | Survey | 306 | Adult | Mixture | 1, 5 |
| Harris, 2014, community, USA | Interviews | 21 | Mean 53, range: 31-78 | Mixture | 4, 5, 6 |
| Harris et al., 2017, community, USA | Interviews | 71 | Mean 53. | Mixture | 5 |
| Harris et al., 2019, community, USA | Interviews | 42 | Mean 50, range: 24-79 | Children | 1, 3, 4, 5 |
| Harris & Levenson, 2021, community, USA | Interviews | 70 | Mean 7, range: 24–79 | Mixture | 1, 3, 4, 5, 6 |
| Harris & Levenson, 2022, community, USA | Focus group | 22 | Mean 48, range 31- 80 | Mixture | 1 |
| Höing et al. 2017, community, Netherlands | Interviews & survey | 17 | Mean 47.9 | Mixture | 2 |
| Höing & Vogelvang, 2013, community, Netherlands | Interviews | 38 | Mean 46.7 | Mixture | 2 |
| Hollomotz, 2021, secure-care, UK | Interviews | 11 | Range 20-60 | Mixture | 2 |
| Ievins & Crewe, 2015, prison, UK | Interviews | 22 | Adults | Mixture | 6 |
| Ievins & Mjåland, 2021, prison, UK | Interviews | 129 | Adults | Mixture | 1, 2, 5 |
| Juventa & Krim, 2017, community, USA | Focus group | 47 | Mean 44. | Mixture | 1, 6 |
| Kemshall et al., 2012, community, UK | Interviews | 61 | Adults | Mixture | 1, 3, 6 |
| Kewley et al., 2017, community, UK | Interviews | 4 | Mean 58, range 50–68 | Children | 5 |
| Kitson-Boyce et al., 2018, prison, UK | Interviews | 9 | Range 45-78 | Mixture | 1, 5, 6 |
| Kitson-Boyce et al., 2019a, community, UK | Interviews | 7 | Range 52-78 | Mixture | 1 |
| Kitson-Boyce et al., 2019b, prison, UK | Interviews | 12 | Range 45-78 | Mixture | 2, 5 |
| Kras et al., 2016, community, USA | Interviews | 98 | Mean 44 | Mixture | 3, 6 |
| Kras, 2019, community and prison, USA | Interviews & file analysis | 72 | Adults | Mixture | 5 |
| Kras, 2022, community and prison, USA | Interviews | 73 | Mean 47, range 21-71 | Mixture | 1, 2, 6 |
| Levenson & Cotter, 2005a, community, USA | Survey | 183 | < 25 (8), 25-64 (86) & >65 (6) | Mixture | 1, 6 |
| Levenson & Cotter, 2005b, community, USA | Survey | 135 | Range 25-64 | Children | 1 |
| Levenson et al., 2007, community, USA | Survey | 239 | <25 (10%), 25-64 (85%), >65 (5%) | Mixture | 1 |
| Levenson, 2008, community, USA | Survey | 109 | Range 26-49 | Mixture | 1 |
| Liem & Weggemans 2018, community, Netherlands | Interviews | 10 | Range 25-65. | Mixture | 1, 3, 4, 5, 6 |
| Lytle et al., 2017, community, USA | Interviews | 25 | Mean age 42.7 | Mixture | 1, 2, 5 |
| Mann et al., 2021, community UK | Interviews | 20 | Range 27-76 | Mixture | 1, 4, 5 |
| McAlinden et al., 2017, community UK | Interviews | 32 | Range 20-80 | Children | 4, 5 |
| McCartan et al., 2021, community, UK | Interviews | 93 | Mean 53, range 24-78 | Mixture | 1, 2 |
| Murphy & Fedoroff, 2013, community, Canada | Interviews | 30 | Adults | Mixture | 1, 6 |
| Paat et al., 2017, community, USA | Interviews | 17 | Adults | Mixture | 1, 6 |
| Page et al., 2012, community, USA | Survey | 231 | 18-33 (37%), 34-41 (37%), 50-65 (19%). | Mixture | 1, 3 |
| Robbers, 2009, community, USA | Survey | 153 | Adults | Mixture | 1, 2, 4, 5, 6 |
| Russell et al., 2013, prison New Zealand | Interviews | 9 | Range 31-63 | Children | 1, 3, 4, 5, 6 |
| Rydberg, 2018, community, USA | Interviews | 65 | Mean 43.8 | Mixture | 3, 4 |
| Sample et al., 2018, community, USA | Interviews | 20 | Adults | Mixture | 2 |
| Sample et al., 2022, community, USA | Interviews | 25 | Adults | Mixture | 2 |
| Sandbukt, 2021, community, Norway | Interviews | 8 | Range 25-80 | Mixture | 4, 5, 6 |
| Schaefer et al., 2004, community, USA | Interviews | 8 | Mean 44, range 36-52 | Children | 4, 5 |
| Seidler, 2010, community, Australia | Interviews | 8 | Adult | Mixture | 1, 6 |
| Simmons et al., 2022, community, USA | Interviews | 14 | Range 50-70 | Mixture | 1, 2, 4, 5, 6 |
| Slater et al., 2023, prison, UK | Interviews | 24 | Mean 39 | Mixture | 4, 6 |
| Spruin et al., 2018, community, UK | Interviews | 25 | Mean 40.1 | Mixture | 1 |
| Ten Bensel & Sample, 2017, community, USA | Interviews | 112 | Mean 41.3 | Mixture | 1, 2, 4 |
| Ten Bensel & Sample, 2019, community, USA | Interviews | 112 | Mean 41.3 | Mixture | 1, 2, 5, 6 |
| Tewksbury & Lees, 2006, community, USA | Interviews | 22 | Mean 48 | Mixture | 1, 4, 5, 6 |
| Tewksbury & Lees, 2007, community, USA | Interviews | 22 | Mean 48 | Mixture | 1 |
| Tewksbury & Connor, 2012, prison, USA | Interviews | 24 | Range 24-67 | Mixture | 5 |
| Tewksbury, 2012, prison, USA | Interviews | 24 | Mean 41, range 24-67 | Mixture | 1, 5 |
| Tewksbury, 2013, community, USA | Interviews | 9 | Mean 38 | Mixture | 1, 4 |
| Tewksbury & Copes, 2013, prison, USA | Interviews | 24 | Range 24-67 | Mixture | 1, 3, 4, 5, 6 |
| Tovey et al., 2022, community, UK | Interviews | 12 | Range 38-65 | Mixture | 1, 4, 6 |
| Vieira et al., 2020, community, UK | Focus group | 6 | Adult | Mixture | 5, 6 |
| Woodall et al., 2013, prison, UK | Interviews & focus group | 36 | Adult | Mixture | 4, 6 |
| Worley & Worley, 2013, community, USA | Interviews | 24 | Mean 44, range 17-66 | Mixture | 1, 6 |
| Youssef et al., 2023, community, Australia | Interviews | 26 | Range 27-74 | Mixture | 1, 2, 4, 5, 6 |
| Zevitz & Farkas 2000, community, USA | Interviews | 30 | Mean 40 | Mixture | 1 -6 |
